# The antiretroviral 2′,3′-dideoxycytidine causes mitochondrial dysfunction in proliferating and differentiated HepaRG human cell cultures

**DOI:** 10.1074/jbc.RA120.014885

**Published:** 2020-12-31

**Authors:** Carolyn K.J. Young, Joel H. Wheeler, Md. Mostafijur Rahman, Matthew J. Young

**Affiliations:** Department of Biochemistry and Molecular Biology, Southern Illinois University School of Medicine, Carbondale, Illinois, USA

**Keywords:** mitochondrial DNA (mtDNA) maintenance, mitochondrial toxicity, 2′-3′-dideoxycytidine (ddC, zalcitabine), nucleoside reverse-transcriptase inhibitor (NRTI), human immunodeficiency virus (HIV), bioenergetics, HepaRG, cell biology, 3TC, 2′,3′-dideoxy-3′-thiacytidine, ANT, adenine nucleotide translocase, APAP or paracetamol, acetaminophen, AZT, 3′ -azido-2′,3′ -dideoxythymidine, CBV, carbocyclic 2′,3′-didehydro-2′,3′-dideoxyguanosine, CO_2_, carbon dioxide, ddC, zalcitabine, 2′-3′-dideoxycytidine, ddCMP, 2′-3′-dideoxycytidine 5′-monophosphate, ddCTP, 2′-3′-dideoxycytidine 5′-triphosphate, ddI, 2′,3′-dideoxyinosine, DIG-11-dUTP, digoxigenin-11-deoxy-uridine-triphosphate, alkali-labile, DPBS, Dulbecco′s phosphate-buffered saline, ECAR, extracellular acidification rate, FCCP, carbonyl cyanide p-trifluoromethoxy-phenylhydrazone, H_2_O, water, MIM, mitochondrial inner membrane, mtDNA, mitochondrial DNA, nDNA, nuclear DNA, NRTI, nucleoside reverse-transcriptase inhibitor, NtRTIs, nucleotide reverse transcriptase inhibitors, OCR, oxygen consumption rate, OXPHOS, oxidative phosphorylation, PBL, peripheral blood lymphocyte, Polγ, mtDNA polymerase gamma, RPTEC, renal proximal tubule epithelial cell, SkMC, skeletal muscle cell, WCE, whole-cell extracted, WDM, Working Differentiation Medium, WGM, Working Growth Medium

## Abstract

Nucleoside reverse transcriptase inhibitors (NRTIs) were the first drugs used to treat human immunodeficiency virus infection, and their use can cause mitochondrial toxicity, including mitochondrial DNA (mtDNA) depletion in several cases. The first-generation NRTIs, including 2′,3′-dideoxycytidine (ddC), were originally and are still pursued as anticancer agents. NRTI-sensitive DNA polymerases localizing to mitochondria allow for the opportunity to poison proliferating cancer cell mtDNA replication as certain cancers rely heavily on mitochondrial functions. However, mtDNA replication is independent of the cell cycle creating a significant concern that toxicants such as ddC impair mtDNA maintenance in both proliferating and nonproliferating cells. To examine this possibility, we tested the utility of the HepaRG cell line to study ddC-induced toxicity in isogenic proliferating (undifferentiated) and nonproliferating (differentiated) cells. Following ddC exposures, we measured cell viability, mtDNA copy number, and mitochondrial bioenergetics utilizing trypan blue, Southern blotting, and extracellular flux analysis, respectively. After 13 days of 1 μM ddC exposure, proliferating and differentiated HepaRG harbored mtDNA levels of 0.9% and 17.9% compared with control cells, respectively. Cells exposed to 12 μM ddC contained even less mtDNA. By day 13, differentiated cell viability was maintained but declined for proliferating cells. Proliferating HepaRG bioenergetic parameters were severely impaired by day 8, with 1 and 12 μM ddC, whereas differentiated cells displayed defects of spare and maximal respiratory capacities (day 8) and proton-leak linked respiration (day 14) with 12 μM ddC. These results indicate HepaRG is a useful model to study proliferating and differentiated cell mitochondrial toxicant exposures.

In human immunodeficiency virus (HIV)-infected patients, nucleoside reverse transcriptase inhibitors (NRTIs) are used to inhibit the reverse transcription of the single-stranded HIV RNA genome into double-stranded DNA. Reverse transcription is carried out by the HIV reverse transcriptase. NRTIs are typically used in combination with other anti-HIV drugs, such as nonnucleotide reverse transcriptase inhibitors, protease inhibitors, integrase strand transfer inhibitors, fusion inhibitors, and chemokine receptor (CCR5) antagonists in scheduled regimens called antiretroviral therapy (1). One potent US Food and Drug Administration–approved NRTI is 2′,3′-dideoxycytidine (ddC), also known as zalcitabine. ddC has been documented to cause unexpected sensorineural deafness, hypertrophic cardiomyopathy, peripheral neuropathy, mitochondrial alterations, and reduced mitochondrial DNA (mtDNA) copy number (2, 3, 4). Nucleotide reverse transcriptase inhibitors (NtRTIs) are generated *in vivo via* phosphorylation of NRTIs by intracellular kinases (5) or occur in a monophosphate bioisostere form (nucleoside phosphonate) such as in the case of tenofovir (PMPA). Nucleoside kinases such as DCK, CMPK1, and nucleoside diphosphate kinases (NME) act on NRTIs, such as ddC, and perform the first, second, and third phosphorylation steps, respectively, generating the metabolically active NtRTI ddCTP in the cytoplasm (6). Once imported into mitochondria, NtRTIs can compete with native nucleotides for DNA polymerase active sites to inhibit mtDNA replication through chain termination. Unlike natural deoxyribonucleotide triphosphate substrates, most NtRTIs lack the 3′ hydroxyl group (3′-OH) and therefore cannot be extended by a polymerase once incorporated into DNA. As a derivative of deoxycytidine, ddC possesses a 3′ hydrogen in place of the deoxycytidine 3’-OH; thus, if ddCMP is not removed from the nascent mtDNA strand *in vivo*, replication will stall. Several DNA polymerases that localize to the mitochondrion, including the replicative mtDNA polymerase gamma (Polγ), are sensitive to inhibition by ddCTP. Anti-HIV NtRTIs including ddCTP, AZT-TP (3′-azido-2′,3′-dideoxythymidine triphosphate), 3TC-TP (2′,3′-dideoxy-3′-thiacytidine triphosphate), D4T-TP (2′,3′-didehydro-2′,3′-dideoxythymidine triphosphate), and carbovir triphosphate (CBV-TP) have been demonstrated to inhibit the nucleotide insertion activity of an exonuclease deficient version of the Polγ p140 catalytic subunit *in vitro*. Furthermore, when a WT version of p140 was separately incubated with DNA substrates containing different 3′-terminal analogs (ddCMP, AZT-MP, 3TC-MP, D4T-MP, or CBV-MP) their exonucleolytic removals were compromised in comparison with control substrates (7). Similarly, the Polγ holoenzyme was demonstrated to remove 3′-end ddCMP, AZT-MP, 3TC-MP, D4T-MP, or CBV-MP from separate primer–template complexes slower than from a control substrate complex harboring a correctly paired dCMP (8, 9). In contrast, the nuclear DNA (nDNA) polymerases alpha (α), delta (δ), and epsilon (ε) harbor strong nucleotide selection mechanisms and are less likely to incorporate NtRTIs (3, 10).

Support for intracellular NRTI/ddC mitochondria toxicity mediated by disruption of mtDNA replication comes from observations that primary and immortalized proliferating cell lines undergo mtDNA depletion upon exposure to various NRTIs (3). In the case of ddC, mtDNA depletion has been observed in primary cell lines such as skeletal muscle cells (1° SkMCs), renal proximal tubule epithelial cells (1° RPTECs), peripheral blood lymphocytes (1° PBLs), as well as cell lines derived from prostate metastatic carcinoma (PC-3), leukemia (MOLT-4 and CEM), hepatocellular carcinoma (HepG2), cervical cancer (HeLa), and other immortalized cell lines (3, 11, 12, 13, 14, 15, 16). Examples of other NRTIs that have been demonstrated to cause reduced mtDNA copy number in cell lines include ddI (2′,3′-dideoxyinosine in CEM, HepG2, 1° SkMCs, 1° RPTECs, and 1° PBLs), 2′,3′-didehydro-2′,3′-dideoxythymidine (in CEM, MOLT-4, HepG2, 1° SkMCs, 1° RPTECs, 1° PBLs, and immortalized human mesenchymal/stromal cell lines, hMSCs), and AZT (in HepG2 and hMSC) (3, 11, 12, 14, 15, 17). The documented NRTI-associated mitochondrial toxicities and resulting manifestations observed in patients have prompted investigations into drug and environmental factors that function as mitochondrial toxicants. Details regarding mitochondrial toxicants have been thoroughly reviewed (3, 18, 19, 20).

Off-target effects of drugs and environmental toxicants causing mitochondrial dysfunction are important factors to consider in toxicity studies, and human cell lines have served as useful models for these experiments (3, 21, 22). The use of *in vitro* human cell culture models in toxicity testing is becoming increasingly attractive owing to the small quantities of compounds needed for testing, shortened experimental timelines, increased throughput to evaluate toxicants, and reduced number and suffering of animals (23, 24). Primary human hepatocytes isolated from liver and liver-derived immortalized cell lines are widely used as models for toxicological studies as the liver is the primary source of drug metabolism and biotransformation (23). The HepaRG cell line was originally derived from a liver tumor obtained from a patient with hepatitis C infection and hepatocarcinoma (25). Genetically, HepaRG has been demonstrated to have a highly stable pseudo-diploid karyotype (25, 26). HepaRG is a proliferating human cell line that can be differentiated into nonproliferating hepatocyte-like and biliary-like cells (27, 28, 29, 30). Differentiated HepaRG cultures have been demonstrated to suffer toxicity from compounds metabolized *via* cytochrome P450s (28). Previously, we utilized HepaRG to gain insight into both undifferentiated proliferating and quiescent differentiated cell metabolism when isogenic cells were exposed to acetaminophen (APAP) and the biguanide metformin (31). We showed that metformin offers protection against loss of APAP-induced cellular viability and prevents APAP-induced decreases in bioenergetics in differentiated but not proliferating-derived HepaRG. It could be distinguished that treatment with metformin alone reduced the mitochondrial bioenergetic parameters ATP-linked respiration, maximal respiratory capacity, and basal respiration in proliferating-derived cells. In addition, we recently performed mtDNA next-generation sequencing and reported the HepaRG mtDNA sequence including its haplogroup branch H15a1 polymorphisms and levels of heteroplasmy (32). In comparison with the revised Cambridge Reference Sequence, HepaRG mtDNA contains 14 nucleotide variations including two heteroplasmic variants, the noncoding control region variant 315insC at 42%, and the novel G13633A *ND5* gene substitution at 33% heteroplasmy.

NRTI-sensitive DNA polymerases localizing to mitochondria afford a unique opportunity to poison proliferating cancer cell mitochondria as certain cancers have an increased reliance on oxidative phosphorylation (OXPHOS), and nDNA polymerases are less sensitive to NRTI inhibition (6, 14). Of interest, nucleoside analogs were originally studied as antimetabolites in the 1950s and the first-generation NRTIs such as ddC, ddI, and AZT were originally pursued as anticancer agents (33, 34, 35). Also, the antibacterial activity of AZT has been reported (36). In 1985 and 1986, these three NRTIs were demonstrated to be anti-HIV agents (37). Different cancer types undergo different bioenergetic alterations with some being more glycolytic and others being more oxidative (38). When treated with ddC, proliferating acute myeloid leukemia cells preferentially activated the analog and blocked mtDNA replication and OXPHOS in comparison with hematopoietic cells (6). Also, ddC was proposed to be used for treating tumors with high levels of expression of DNA polymerase beta (Polβ) (39). Furthermore, the combined use of AZT and ddI was shown to reduce tumor growth of human colorectal carcinoma HCT-116 cells in mice (40). As mammalian mtDNA replication takes place independently from the cell cycle (41, 42, 43, 44, 45) mtDNA maintenance disrupting drugs, such as ddC, will likely impair mitochondrial functions in both proliferating and nonproliferating cells. Furthermore, studying the effect of ddC on different cell types is necessary, as the bioenergetic consequence of ddC on quiescent differentiated cells has not been thoroughly investigated. Evaluation of the off-target effects of drugs on mitochondrial function using human cell lines is an important consideration for determining mitochondrial toxicity because the complex mitochondrial network harbors multiple copies of OXPHOS complexes and mtDNA that may cause a slow response to these agents. It must also be noted that HIV-infected patients have undergone chronic months-long treatments with ddC (2). To gain information about the mechanism of ddC mitochondrial toxicity, here we examine the effects of ddC exposure on proliferating and nonproliferating/differentiated HepaRG cell viability, mtDNA maintenance, and mitochondrial bioenergetics.

## Results

### Differentiated HepaRG cells have higher levels of mtDNA than proliferating cells

**Figure 1:**
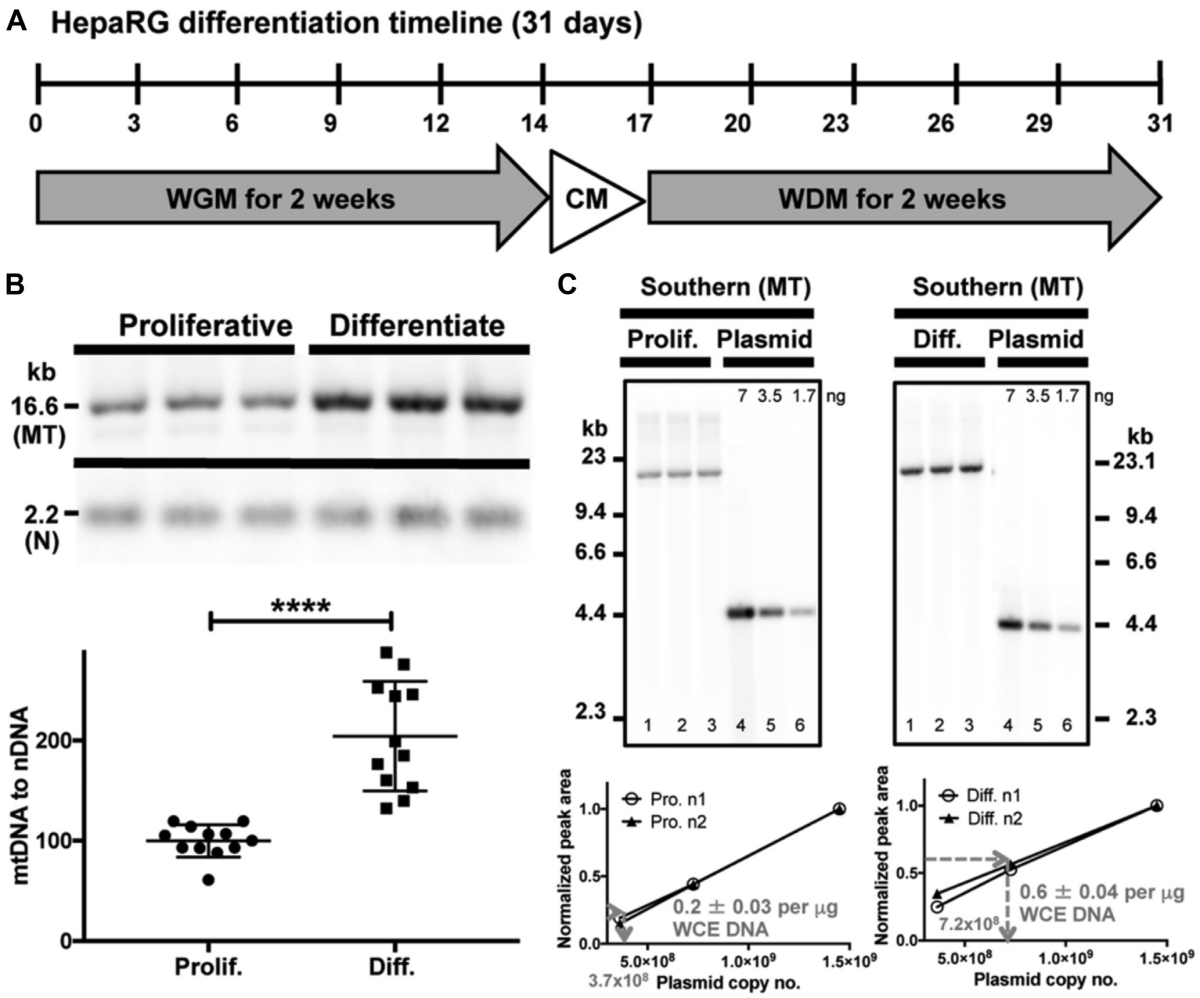
**HepaRG differentiation timeline and mitochondrial DNA (mtDNA) content.***A*, timeline of HepaRG differentiation. CM, Combination Medium; WDM, Working Differentiation Medium; WGM, Working Growth Medium (see Experimental procedures). *B*, a representative Southern blot of relative proliferating and differentiated HepaRG mtDNA content. Triplicate 1 μg reactions of whole-cell extracted (WCE) DNA were digested with BamHI, then loaded and electrophoresed on an agarose gel before blotting. The Southern blot was probed simultaneously with mtDNA (MT) and nDNA (N) probes. Proliferating HepaRG WCE DNA samples were prepared from cells obtained 7 days post-seeding at 2 × 10^4^ cells/cm^2^. Differentiated HepaRG DNA samples were prepared from cells obtained 7 days post-differentiation. BamHI-digested human genomic DNA samples generate a 16.6-kb mtDNA genome-length band and a 2.2-kb nDNA band as described (79). Using the relative comparison of the MT to the N probe, differentiated HepaRG cells contain ∼2-fold more mtDNA than proliferating cells. Data are presented as mean ± standard deviation (SD); n = 12 from n ≥ 3 whole-cell DNA extract preparations derived from independent passages. Four Southern blots containing triplicate sample replicates were used for the analysis. For each lane of the dual-probed blots, the mtDNA peak 'Percent' value was normalized to the 18S nDNA peak 'Percent' value. A peak percent value is defined as the area of a peak measured as a percent of the total size of all measured peak areas on a blot. The mean normalized band intensity values of the proliferating HepaRG samples were set to 100% (∗∗∗∗*p* < 0.0001). *C*, quantitation of mtDNA molecules per cell. Representative blots are shown for each of proliferating (Prolif.) and differentiated (Diff.) HepaRG. Triplicate 1 μg reactions of WCE DNA were digested with BamHI then loaded and electrophoresed on an agarose gel. In parallel, 7.0, 3.5, and 1.7 ng of HindIII linearized pCR2.1-TOPO-mtDNA plasmid (4.4-kb) were loaded. The blots were detected with the MT probe. Three-point standard curves based on the pCR2.1-TOPO-mtDNA peak area percent values were used to estimate the mtDNA copy number and the 7-ng plasmid bands were set to 1. Two curves from two blots are plotted below the representative blots. The average mtDNA peak area percent values for 1 μg proliferating or differentiated HepaRG whole-cell DNA extracts are shown in gray with errors reported as SD (n = 6; triplicate measurements from two independent experiments using different passages; P10 and P14, differentiated; P8 and P9, proliferating HepaRG). On average, 1 μg of proliferating HepaRG WCE DNA was prepared from 1.4 × 10^5^ ± 4.4 × 10^4^ cells and 1 μg of differentiated WCE DNA was prepared from 9.8 × 10^4^ ± 1.7 × 10^4^ cells (n = 6 for each; triplicate cell counts from two independent experiments using different passages). The calculated copies of mtDNA are 3.7 × 10^8^ mtDNA/μg or 2600 mtDNA genomes per proliferating cell and 7.2 × 10^8^/μg or 7400 mtDNA genomes per differentiated cell.

As mammalian mtDNA replication is independent of the cell cycle we wanted to determine mtDNA levels in both proliferating and differentiated HepaRG cell phases (41, 42, 43, 44, 45). DNA samples from both cell types were compared using Southern blotting and dual digoxigenin (DIG)-labeled probe hybridization to estimate mtDNA content. Proliferating HepaRG whole-cell extracted (WCE) DNA samples were prepared from cells obtained at 7 days post-seeding at 2 × 10^4^ cells/cm^2^, and differentiated HepaRG DNA samples were prepared from cells harvested at 7 days post-differentiation, Figure 1*A*. The levels of mtDNA in each lane of the blot were detected with the mtDNA-specific probe and were normalized to nDNA levels detected with the 18S probe in the same lane, Figure 1*B*. Using this relative comparison method, differentiated HepaRG contains ∼2-fold more mtDNA compared with proliferating cells. Since differentiated cells are no longer undergoing cell division these cells likely maintain a higher level of mtDNA copy number that is necessary for mitochondrial bioenergetic functions.

Next, we determined the number of mtDNA molecules in WCE DNA samples using Southern blotting and the mtDNA DIG-labeled probe. Three-point standard curves of known quantities of linearized pCR2.1-TOPO-mtDNA plasmid harboring the mtDNA template sequence used to generate the mtDNA probe were loaded onto gels in parallel with WCE DNA samples. The number of mtDNA molecules in proliferating and differentiated HepaRG WCE DNA samples was calculated utilizing linear regression analysis of plasmid band intensities (peak area percent values), Figure 1*C*. In good agreement with the dual probe (relative comparison) method, we calculated there are at least 2600 and 7400 mtDNA genomes per proliferating and differentiated HepaRG cell, respectively (2.9-fold more mtDNA per differentiated cell). By comparison, using a dot blot hybridization technique, human 143B cybrid cell lines were reported to harbor an average of 13,000 wild-type mtDNA genomes per cell (46).

### Proliferating HepaRG cell growth is inhibited by exposure to ddC

**Figure 2:**
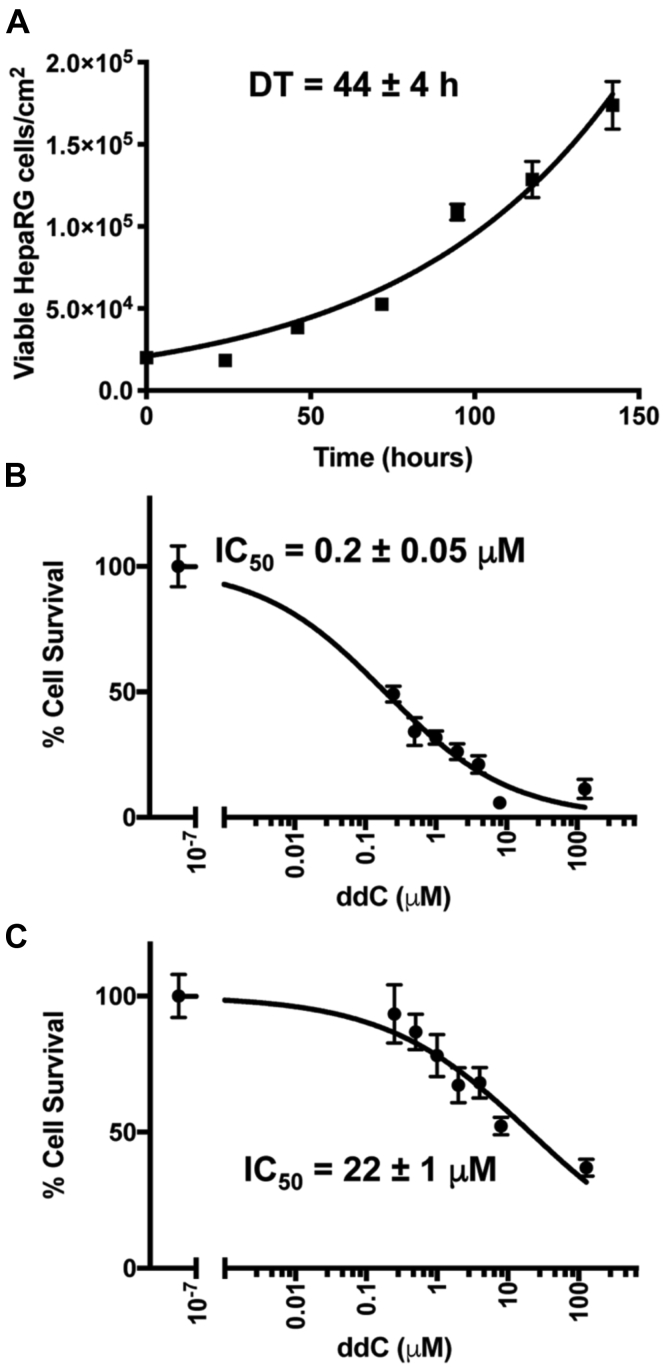
**HepaRG growth rate and survival following 2′,3′-dideoxycytidine (ddC) treatment.***A*, proliferating HepaRG growth rate was determined by growing cells in Working Growth Medium (WGM) at 5% CO_2_, 37 °C. Cells were seeded at 2 × 10^4^ cells/cm^2^. The mean doubling time (DT) based on three independent experiments utilizing HepaRG at passages P8, P9, and P16 is reported in hours (h) with error as SD. Mean viable cell count values (n = 4) and SDs for a representative experiment (P8) are shown in the graph. DT values were calculated using the least-squares fit of the exponential growth equation in Graph Pad Prism. *B*, a representative proliferating HepaRG survival curve is shown following exposure to ddC. The mean IC_50_ value based on two independent experiments utilizing HepaRG at passages P15 and P16 is reported in micromolar (μM) with error as SD. Cells were seeded at 2 × 10^4^ cells/cm^2^ in WGM the day before the addition of WGM treatment media. *C*, a representative differentiated HepaRG survival curve is shown following exposure to Working Differentiation Medium ddC treatment media. The mean IC_50_ value based on two independent experiments utilizing differentiated cells at passages P8 and P10 is reported in micromolar with error as SD. In *B*, proliferating and *C*, differentiated experiments, cells were exposed to 128, 8, 4, 2, 1, 0.5, 0.25, and 0 μM ddC for 15 days, and mean survival values (n ≥ 3) and SDs are reported for each graph. IC_50_ values were calculated using the least-squares fit of inhibitor concentration *versus* normalized response in Graph Pad Prism.

Cell survival was determined by counting the number of viable cells utilizing the trypan blue exclusion method. The doubling time of proliferating HepaRG under standard tissue culture conditions is 44 ± 4 h; the error reported is the standard deviation (SD), Figure 2*A*. Assuming that during ddC treatment the proliferating cells continue to divide, mtDNA replication is blocked, and that following a cell division parental cell mtDNA (2600 mtDNA/cell) is equally distributed among daughter cells, then after 15 days of treatment, we would expect mtDNAs per cell to be <1% of initial levels. Here we examined a range of concentrations from 0 to 128 μM that included 0.5 and 1 μM ddC, as peak plasma concentrations of 0.5 and 1 μM ddC have been reported in patients (47, 48), Figure 2, *B*–*C*. Of interest, ddC likely accumulates at different concentrations within different organs and tissues as indicated by an experiment using rats where differences in unbound ddC distribution have been measured. Rat muscle contained ∼8-fold higher concentrations of ddC compared with the brain, 238 and 31 μM, respectively (49). We determined the half-maximal inhibitory concentration (IC_50_) value or concentration of ddC, which reduces the number of viable treated cells by 50% relative to untreated control cells. Following 15 days of ddC treatment, we observed the recognizable symmetrical sigmoidal shape of log(toxicant) *versus* percent cell survival for proliferating HepaRG, Figure 2*B*. When examining the number of live cells remaining post-treatment and normalizing to untreated cells, proliferating HepaRG growth and survival was inhibited by 50% at 0.2 ± 0.05 μM ddC. This value is similar to the IC_50_ values reported for human THLE-2 liver epithelial cells after 6 days of treatment with ddC, 1.6 ± 2 μM (50). In contrast, differentiated HepaRG cells are significantly less sensitive to ddC having an IC_50_ of 22 ± 1 μM, Figure 2*C*.

### Experimental design for determining the effects of ddC on mitochondrial functions

**Figure 3:**
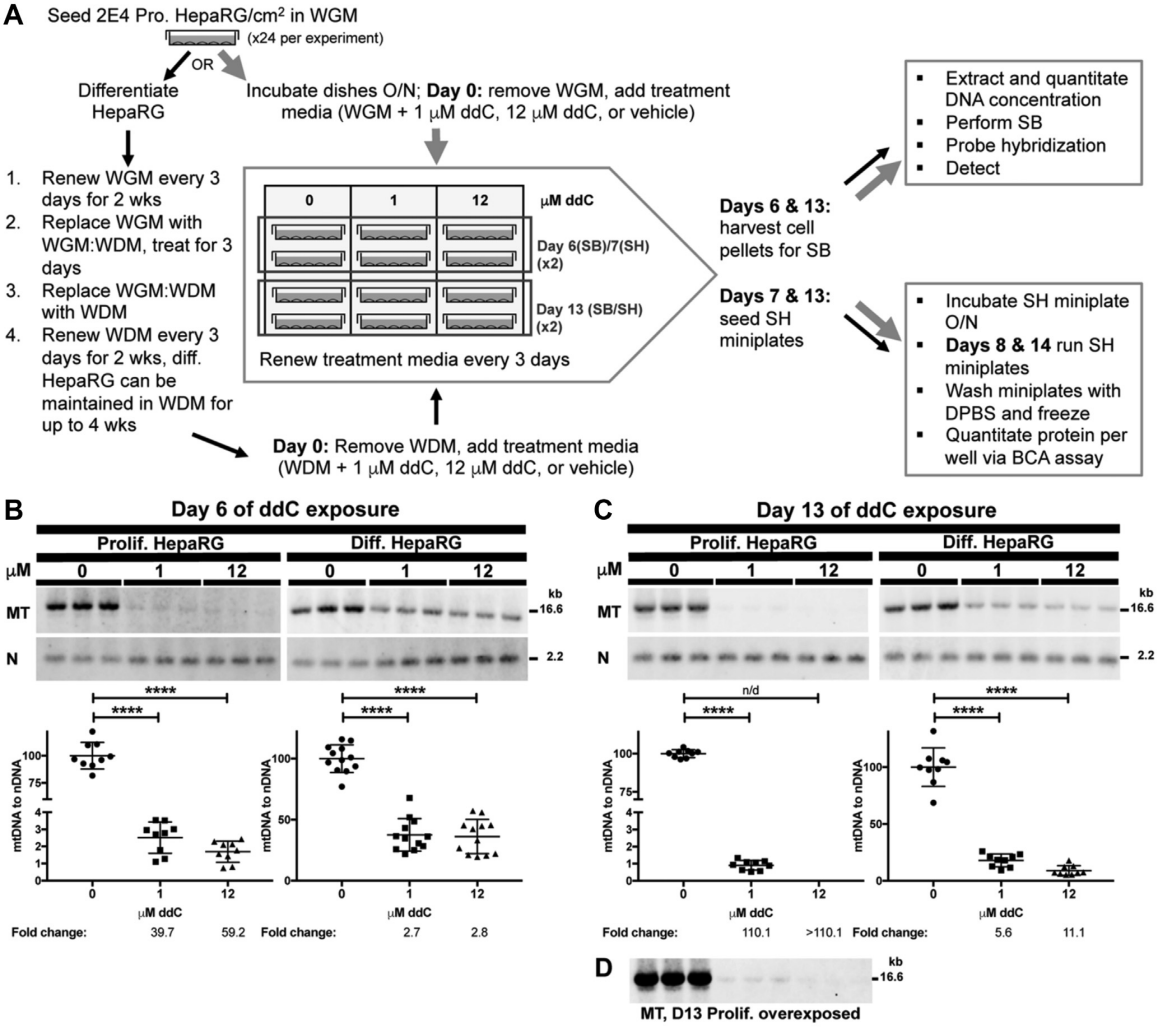
**A greater amount of mitochondrial DNA (mtDNA) depletion is observed in proliferating HepaRG as compared with differentiated HepaRG after 2′,3′-dideoxycytidine (ddC) exposure.***A*, workflow for seeding, culturing, and exposing HepaRG cells to ddC. Twenty-four tissue culture dishes were seeded with 2 × 10^4^ proliferating HepaRG cells/cm^2^, then either subjected to the differentiation protocol (*thin black arrows*) or incubated overnight (O/N), then exposed to ddC on the next day in Working Growth Medium (WGM) (*thick gray arrows*). After the 31-day differentiation process, cells were exposed to ddC in Working Differentiation Medium (WDM), Day 0. For each experiment, one-quarter of the tissue culture dishes were harvested on day 6 post-ddC exposure for Southern blotting (SB) and one-quarter were harvested on day 7 for Seahorse (SH). The remaining half of the tissue culture dishes were harvested on day 13. Experiments were repeated three times with three different cell passages (see Experimental procedures for details). Southern blots of proliferating and differentiated HepaRG mtDNA content following *B*, 6 and *C*, 13 days of ddC exposure. A representative blot is shown for each experiment. Triplicate 1 μg reactions of whole-cell DNA extracts were digested with BamHI then loaded and electrophoresed on an agarose gel before blotting and nonradioactive probe hybridization. Except for blots containing samples obtained from proliferating HepaRG at day 13, mtDNA content was measured on blots that were simultaneously probed with the 18S nDNA probe (N) and the mtDNA-specific probe (MT) as described in the legend for Figure 1, and the mean normalized band intensity values of the vehicle control samples (0 μM ddC) were set to 100%. Dual-probed blots containing day 13 samples from proliferating HepaRG treated with 1 μM could not be quantitated owing to low mtDNA signal; however, when the blots were stripped and probed individually with the mtDNA probe, stripped again, and then probed with the 18S probe, 0 and 1 μM mtDNA bands could be quantitated in Fiji as described (79). To confirm equal loading of whole-cell extracted DNA into each lane, the %CV for the nDNA bands on the single-probed 18S blots were at most 11% (100% ± 11% for 0 μM; 102% ± 10% for 1 μM; 101% ± 8% for 12 μM, errors are SDs), and the 0, 1, and 12 μM ddC–treated 18S sample band intensities are not significantly different from one another as judged by a one-way ANOVA. Data presented in the graphs are mean ± SD, n ≥ 9 (≥3 blots from three independent experiments using different preparations/passages of cells); statistically significant differences in mtDNA content were determined by ANOVAs except for proliferating HepaRG day 13, which was done by a Welch’s *t* test as the 12 μM ddC–treated sample had no detectable mtDNA. ∗∗∗∗*p* ≤ 0.0001 between vehicle control (0 μM) and 1 or 12 μM. *D*. An overexposed image of proliferating HepaRG day 13 samples with the mtDNA-specific probe (same image as in *C*). BCA, bicinchoninic acid.

Previous experiments examining the off-target effects of ddC on mitochondrial functions have exploited proliferating human cell lines, varying incubation times (3–15 days), and varying concentrations of ddC, *e.g.*, 0.05, 0.5, 5, 10, 20, and 30 μM (3, 11, 14, 15, 16, 51, 52). Based on our survival curve experiments, the reported peak concentrations of ddC in plasma, and the chronic months-long dosing periods reported for HIV-infected patients (2), we chose to examine 1 and 12 μM ddC to determine the effects of ddC on proliferating and differentiated HepaRG mtDNA maintenance, cellular viability, and mitochondrial bioenergetics. At time point zero (day 0) ddC was added to either Working Growth Medium (WGM) or Working Differentiation Medium (WDM), then proliferating or differentiated HepaRG cells were exposed to the WGM + ddC or WDM + ddC treatment media, respectively. One quarter of the cells were harvested on day 6 for Southern blot analysis of WCE DNA and one quarter were harvested on day 7 for bioenergetic analysis. The remaining half of the cells were harvested on day 13, Figure 3*A*.

### Treatment with ddC causes rapid mtDNA depletion in proliferating HepaRG

As a proliferating HepaRG cell contains 2.9-fold less mtDNA than a differentiated cell, we predicted that mtDNA depletion would be more severe for proliferating cells following exposure to ddC. Southern blotting demonstrated extreme depletion of proliferating HepaRG mtDNA relative to nDNA for both 1 and 12 μM ddC–treated cells at 6 and 13 days post-treatment, Figure 3, *B*–*C*. Relative to untreated control cells, after 6 days of exposure to 1 and 12 μM ddC proliferating HepaRG retained only 2.5% and 1.7% mtDNA, respectively, Figure 3*B*. After 13 days of exposure to 12 μM ddC, mtDNA levels were so low that they could not be measured, Figure 3, *C*–*D*. And, after 13 days of exposure to 1 μM ddC, mtDNA levels dropped to 0.9% of untreated HepaRG mtDNA levels. These data support that proliferating HepaRG mtDNA is sensitive to rapid depletion upon exposure to ddC.

### Treatment with ddC causes slower mtDNA depletion in differentiated HepaRG

Southern blotting revealed that ddC treatment caused a slower rate of mtDNA depletion in differentiated HepaRG relative to proliferating cells, Figure 3, *B*–*C*. Relative to untreated control cells, after 6 days of exposure to 1 and 12 μM ddC, differentiated cells harbored 37% and 36% of untreated HepaRG mtDNA levels, respectively, Figure 3*B*. After 13 days, mtDNA levels fell to 18% and 9% for 1 and 12 μM ddC–treated cells, respectively, Figure 3*C*. Based on these data, we estimate mtDNA depletion due to ddC exposure to occur 15× to 21× faster in proliferating *versus* differentiated HepaRG (*e.g.*, for differentiated to proliferating HepaRG at 1 μM ddC day 6, 37%/2.5% = 15). If differentiated cells did not undergo mtDNA replication, then one would expect that ddC treatment would not affect mtDNA copy number; thus, these data support the concept that mtDNA replicates independently from the cell cycle in quiescent and nonreplicating postmitotic cells (41, 42, 43, 44, 45).

### Only proliferating HepaRG viability is reduced following a 13-day ddC exposure

**Figure 4:**
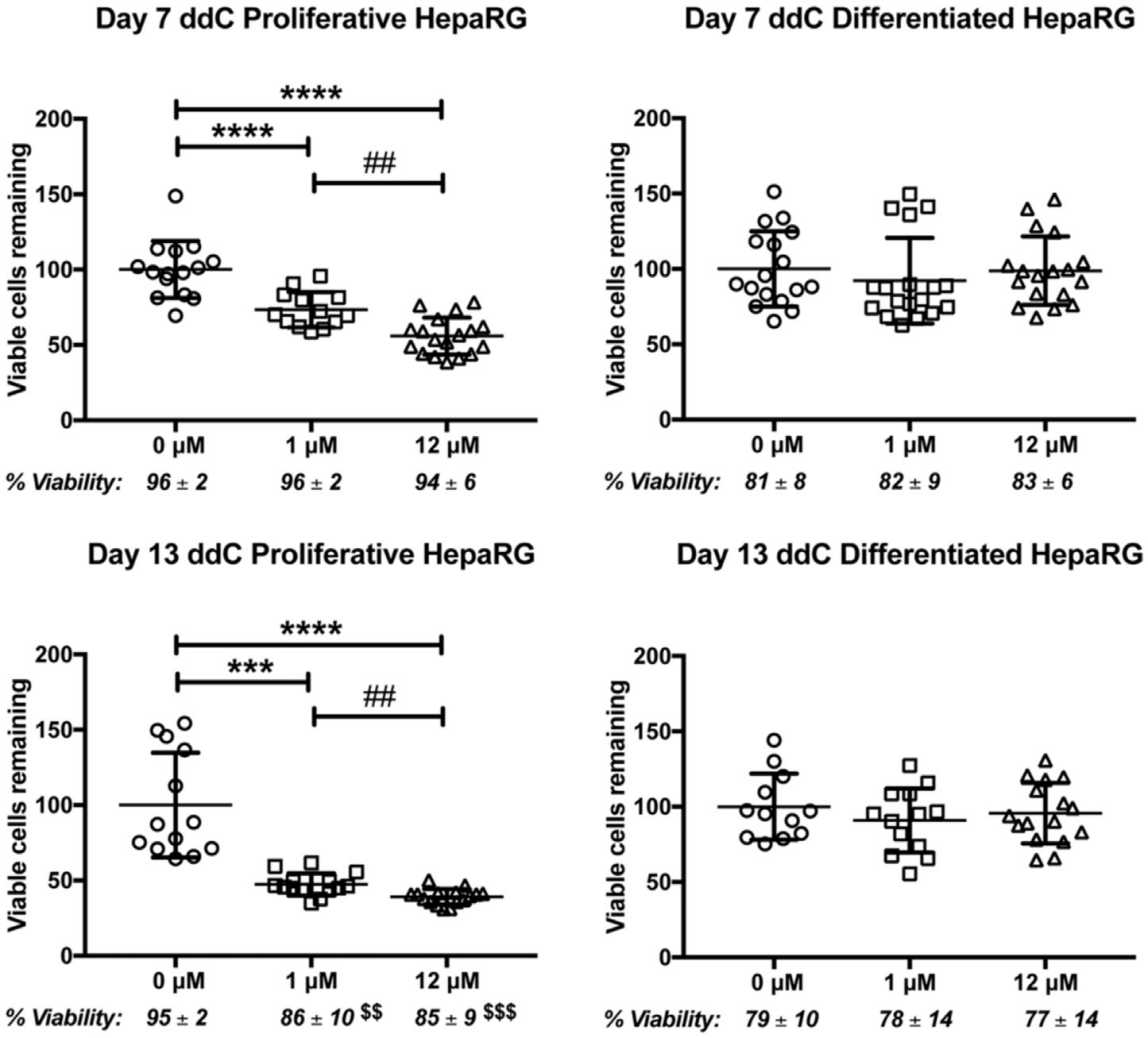
**HepaRG viable cell counts following 2′,3′-dideoxycytidine (ddC) exposure.** HepaRG cells were exposed to vehicle, 1, or 12 μM ddC for 7 and 13 days, then cells were counted. The number of viable cells remaining on the cell culture dishes was determined utilizing the trypan blue exclusion method. In each set of experiments, the vehicle control–treated (0 μM ddC) cell counts were set to 100 (%). Data are presented as mean viable cell counts ±SD, n ≥ 12 (≥quadruplicate from three independent experiments using different cell passages). The percentage of the viable (clear) cells of the total remaining cell population (blue and clear) is reported under the graphs as percent viability. The statistical significance was determined by one-way ANOVA for parametric data and by Kruskal–Wallis tests for nonparametric data. ∗∗∗∗*p* ≤ 0.0001 between vehicle control (0 μM) and 1 or 12 μM; ∗∗∗*p* ≤ 0.001 between 0 and 1 μM; ##*p* ≤ 0.01 between 1 and 12 μM.

Proliferating and differentiated HepaRG cell counts and viability were measured on days 7 and 13 post-ddC exposure utilizing the trypan blue exclusion method. Proliferating HepaRG cells displayed reductions in the number of viable cells remaining on cell culture dishes that correlated with increasing ddC concentration and treatment time, Figure 4. In comparison with vehicle control–treated viable cell counts (100%), by exposure day 7, 73.4% and 55.8% of the 1 and 12 μM ddC–treated viable cells remained on the dishes, respectively. Six days later (day 13 post-ddC exposure), the cell counts were 47.4% and 39.1% of the control cell counts for the 1 and 12 μM ddC–treated proliferating cells, respectively. The viability of the proliferating cell population remaining on the culture dishes remained high (≥94%) for proliferating cells treated with 0, 1, and 12 μM ddC over 7 days. However, at 13 days post-treatment relative to control-treated cells, the viability of the remaining cells on the dish for 1 and 12 μM ddC treatments decreased to 86% and 85%. Simply put, proliferating HepaRG cell counts and viability deteriorated with lengthier ddC exposure. In contrast, differentiated cells maintained vehicle control levels of percent viability at both concentrations of ddC and at both time points. Differentiated HepaRG cells exposed to 1 and 12 μM ddC displayed trends of slight reductions in the number of viable cells remaining on cell culture dishes; however, these trends were not significantly different from vehicle control–treated cell counts, Figure 4.

### Exposure to ddC causes severe deterioration of proliferating HepaRG bioenergetics

To determine mitochondrial bioenergetic profiles, cells were exposed to known pharmacological stressors of the OXPHOS machinery. Mitochondrial OXPHOS is the O_2_-dependent process of coupling substrate oxidation to the production of the energy-rich molecule adenosine triphosphate (ATP). During OXPHOS, molecular O_2_ is reduced to water (H_2_O). An XFp O_2_ biosensor measures the real-time rate at which cells convert O_2_ to H_2_O, the O_2_ consumption rate (OCR). A second XFp biosensor measures the extracellular acidification rate (ECAR) resulting from the cytoplasmic breakdown of glucose-derived pyruvate to lactate and the respiratory evolution of carbon dioxide (CO_2_). Glycolysis is the major cytosolic O_2_-independent metabolic pathway that converts one molecule of glucose into two molecules of pyruvate, ATP, and NADH. When pyruvate is shunted through the mitochondrion to the pyruvate dehydrogenase complex and subsequently through the tricarboxylic acid cycle, CO_2_ is generated. In solution, a molecule of CO_2_ can combine with a molecule of H_2_O forming carbonic acid that dissociates at physiological pH into the bicarbonate anion and a proton that contributes to medium acidification (53).

**Figure 5:**
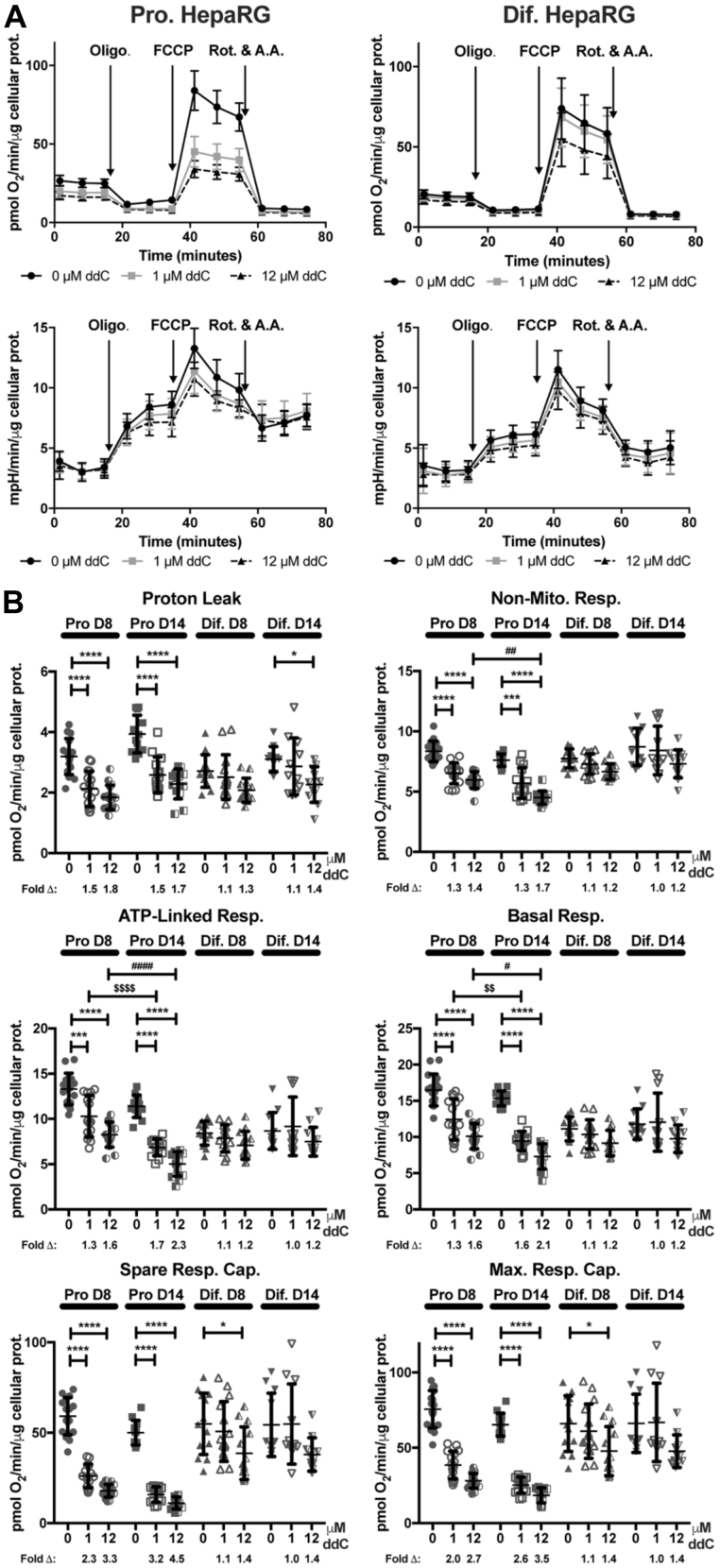
**Exposure to 2′,3′-dideoxycytidine (ddC) causes aberrant bioenergetics in proliferating and differentiated HepaRG cells.***A*, proliferating (Pro.) and differentiated (Dif.) HepaRG Mito Stress test oxygen consumption rates (pmol O_2_/min/μg cellular protein) and extracellular acidification rates (mpH/min/μg cellular protein) following 8 days of treatment with 0, 1, or 12 μM ddC. Metabolic stressors were injected sequentially from Ports *a* (2 μM oligomycin final well concentration), *b* (1 μM FCCP final well concentration), and *c* (0.5 μM antimycin A + 0.5 μM rotenone, final well concentrations). A.A., antimycin A; Olig., oligomycin; Rot., rotenone. Data are mean values ± SD; n ≥ 12, ≥quadruplicate from three independent experiments using different preparations/passages of cells. *B*, scatter dot plots of proliferating and differentiated HepaRG mitochondrial bioenergetic parameters following 8 and 14 days of ddC exposure. Bioenergetic parameters: Proton Leak, and nonmitochondrial respiration (Non-Mito Resp.), ATP-linked respiration (ATP-linked Resp.), basal respiration (Basal Resp.), spare respiratory capacity (Spare Resp. Cap.), and maximal respiratory capacity (Max. Resp. Cap.). Data are presented with mean values and SD errors on the plots, n ≥ 11 (greater than triplicate from three independent experiments using different passages). The fold-changes (Δ) of 0 μM ddC (control)-treated bioenergetic parameters relative to 12 and 1 μM ddC–treated cells are listed under each plot. The statistical significance was determined by three-way ANOVA. ∗∗∗∗*p* ≤ 0.0001, 0 *versus* 1 or 12 μM; ∗∗∗*p* ≤ 0.001, 0 *versus* 1 μM; ∗*p* ≤ 0.05, 0 *versus* 12 μM; $$$$*p* ≤ 0.0001, D8 *versus* D14 at 1 μM; $$*p* ≤ 0.01, D8 *versus* D14 at 1 μM; ####*p* ≤ 0.0001, D8 *versus* D14 at 12 μM; ##*p* ≤ 0.01, D8 *versus* D14 at 12 μM; #*p* ≤ 0.05, D8 *versus* D14 at 12 μM. The *p*-values for differentiated HepaRG spare respiratory and maximal respiratory capacities on day 14 post-treatment with 12 μM ddC were 0.07 and 0.09, respectively. Bioenergetic parameters were calculated as previously described (31).

To determine the effects of ddC on HepaRG bioenergetic profiles, Mito Stress tests were conducted using the Seahorse XFp. Oligomycin, FCCP, and rotenone plus antimycin A were sequentially injected during the experiments, Figure 5 *A*. Rotenone and antimycin A are inhibitors of OXPHOS complexes I and III, respectively, and were the last stressors added during the experiments to terminate electron flow through the electron transport chain and to enable the calculation of oxygen consumption from nonmitochondrial oxidases. This *nonmitochondrial respiration* parameter represents cellular OCR remaining following rotenone plus antimycin A treatment (see Fig. S1). The *basal respiration* parameter is the difference between the OCR measured just before oligomycin injection and the nonmitochondrial respiration parameter. Basal respiration represents the sum of the respiration used to power ATP production and respiration associated with proton leakage across the inner membrane. Following the injection of oligomycin to inhibit complex V, ATP production slows and respiration utilized to power ATP production decreases. The amount of basal OCR sensitive to oligomycin represents a measurement of *ATP-linked respiration* (53). After subtracting the nonmitochondrial respiration rate, FCCP stimulation of OCR provides a measurement of the *maximal respiratory capacity* of cells and by extension the maximal capacity to oxidize substrates *via* the OXPHOS machinery. Next, the difference between the maximal respiratory capacity and basal respiration was used to calculate the *spare respiratory capacity*. Spare respiratory capacity is defined as the extramitochondrial capacity available to produce ATP during stress or increased work. The spare respiratory capacity is suggested to be important for cellular function and long-term survival (54). During substrate oxidation, a portion of the proton motive force is not utilized to drive ATP synthesis *via* complex V, and some protons “leak” back into the matrix *via* inducible uncoupling proteins and through the presence (not the activity) of the inner membrane adenine nucleotide translocase, ANT (55). This *proton leak–linked respiration* parameter is the remaining mitochondrial respiration detected in the presence of oligomycin.

We examined the effects of cell type (proliferating or differentiated), time (day 8 or 14), and ddC concentration (0, 1, and 12 μM) on bioenergetic parameters. We begin by describing proliferating cell results and highlight the OCR and ECAR profiles following 8 days of 0, 1, or 12 μM ddC exposure to illustrate the effect of the NRTI on HepaRG OCRs and by extension mitochondrial function, Figure 5*A*. The effects of day 8 and 14 ddC exposures on proliferating and differentiated HepaRG bioenergetic parameters are shown in Figure 5*B*.

Proliferating HepaRG mitochondria were sensitive to 8-day ddC exposures. Following the 1 and 12 μM ddC treatments proliferating cells had the greatest defects in spare and maximal respiratory capacities, Figure 5*B*. For 1 μM ddC–treated cells, spare respiratory and maximal respiratory capacities were 2.3- and 2.0-fold reduced from vehicle control–treated cells, respectively. For 12 μM ddC–treated proliferating cells, spare and maximal respiratory capacities were 3.3- and 2.7-fold reduced. For 1 μM ddC–treated cells, proton leak–linked respiration was reduced 1.5-fold and nonmitochondrial respiration, basal respiration, and ATP-linked respiration were the least changed of the various parameters having 1.3-fold reductions from vehicle control; however, these changes are all significantly different. Similar trends were noted for 12 μM treated cells with greatest defects ranging from spare respiratory capacity (3.3-fold) > maximal respiratory capacity (2.7-fold) > proton leak–linked respiration (1.8-fold) > ATP-linked respiration (1.6-fold) = basal respiration (1.6-fold) > nonmitochondrial respiration (1.4-fold reduced from control).

After six more days of ddC exposure (day 14 post-treatment) proliferating HepaRG bioenergetic parameters including basal, ATP-linked, and nonmitochondrial respiration continued to crash, Figure 5*B*. Significant differences in day 8 *versus* day 14 basal and ATP-linked respirations were observed between cells treated with both 1 and 12 μM ddC. Compared with control-treated cells, the basal respiration for 1 and 12 μM ddC treatments dropped from 1.3- and 1.6-fold on day 8 to 1.6- and 2.1-fold reductions on day 14, respectively. Likewise, the ATP-linked respiration for 1 and 12 μM ddC–treated cells fell from 1.3- and 1.6-fold on day 8 to 1.7- and 2.3-fold on day 14. For nonmitochondrial respiration, only the 12 μM treated samples were significantly different between days 8 and 14 decreasing from 1.4- to 1.7-fold, respectively.

Similar to what was observed at 8 days post-treatment, after 14 days of ddC exposure the 1 and 12 μM treated proliferating cells had the greatest defects (fold-changes) in spare and maximal respiratory capacities. For 1 μM ddC–treated cells, spare and maximal respiratory capacities were 3.2- and 2.6-fold reduced from vehicle control–treated cells, respectively. For 1 μM ddC–treated cells, proton leak–linked and nonmitochondrial respiration were decreased by 1.5- and 1.3-fold, respectively. The range of bioenergetic defects for 12 μM treated cells at day 14 from highest to lowest were spare respiratory capacity (4.5-fold) > maximal respiratory capacity (3.5-fold) > ATP-linked respiration (2.3-fold) > basal respiration (2.1-fold) > proton leak–linked respiration (1.7-fold) = nonmitochondrial respiration (1.7-fold reduced from control).

### Exposure to ddC causes milder deterioration of differentiated HepaRG bioenergetics

Although several differentiated HepaRG bioenergetic parameters were slightly reduced upon 1 μM ddC treatment following 8 and 14 days of exposure (day 8: proton leak, basal respiration, and nonmitochondrial respiration; day 14: proton leak) these changes, as well as the other bioenergetic parameters, were not significantly different from control-treated cells, Figure 5*B*. Similarly, upon 12 μM ddC treatment following 8 and 14 days of exposure many parameters were slightly reduced but the decreases were not significant (day 8: proton leak, ATP-linked respiration, basal respiration, nonmitochondrial respiration; day 14: spare respiratory capacity, maximal respiratory capacity, ATP-linked respiration, basal respiration, nonmitochondrial respiration). Significant changes were observed for 12 μM ddC treatments at day 8 including spare respiratory and maximal respiratory capacities. Both capacities were 1.4-fold reduced from control-treated cells. Finally, by day 14, 12 μM ddC–treated differentiated cell proton leak–linked respiration was 1.4-fold reduced from control-treated cells, Figure 5*B*. Taken together with the mtDNA depletion, percent viability, and chronic/long-term treatment of patients with NRTIs, these results emphasize the importance of understanding the antiviral off-target effects on mitochondrial functions in different cell types.

## Discussion

Recently, Southern blotting served as a powerful tool to characterize and identify the human degradosome, the mitochondrial machinery that degrades mtDNA (56). In the degradosome model, the p140 catalytic subunit of Polγ harboring the 3′-5′ exonuclease activity, the 5′-3′ mtDNA Twinkle helicase, and the 5′-3′ exonuclease MGME1 work in concert to quickly degrade linear mtDNA molecules. We predict that mtDNA genome instability in nonproliferating differentiated cells is initiated *via* ddC-stalled replisomes. In this scenario, the mtDNA degradosome machinery degrades partially synthesized and stalled nascent strands and broken mtDNA strands leading to mtDNA depletion and loss of mitochondrial bioenergetics. Based on our quantitative analysis of 7400 mtDNA molecules per differentiated HepaRG cell (Fig. 1) and assuming a steady-state rate of mtDNA synthesis and degradation in noncycling cells, we estimate that, on the 13th day of 12 μM ddC exposure the 9% level of mtDNA (11.1-fold, Fig. 3*C*) represents ∼700 mtDNA molecules per cell. This represents a loss of ∼22 mtDNA molecules per hour (depletion of 7400 − 700 = 6700 molecules over 312 h = ∼22). By the eighth day of 12 μM ddC treatment, the differentiated HepaRG spare respiratory and maximal respiratory capacity bioenergetic parameters were slightly but significantly reduced compared with control cells, Figure 5*B*. These reductions are predicted to weaken the ability of differentiated cells to respond to increased energy demands and to withstand stressful conditions (53). Likewise, following 14 days of 12 μM ddC treatment, proton leak–linked respiration was reduced 1.4-fold. Thus, the results of this study support that mitochondrial genotoxic agents impair nonproliferating cell mitochondrial functions. Several proteins associate with mtDNA at distinct nucleoid structures on the matrix-side of the mitochondrial inner membrane (MIM) (57). Mitochondrial nucleoids occur at specific voids between mitochondrial cristae (58) suggesting mtDNA-containing nucleoids could provide a structural role at the MIM that is necessary for the proper function of membrane proteins. As some protons leak back into the matrix *via* the MIM-localized ANT, and ANT2 (*SLC25A5*) and ANT3 (*SLC25A6*) have been identified as human mtDNA nucleoid proteins (55, 59), we predict that ddC-induced mtDNA depletion in differentiated HepaRG could alter the structure of the MIM and the MIM-localized ANT gated pores resulting in decreased proton leakage through aberrant pores.

**Figure 6:**
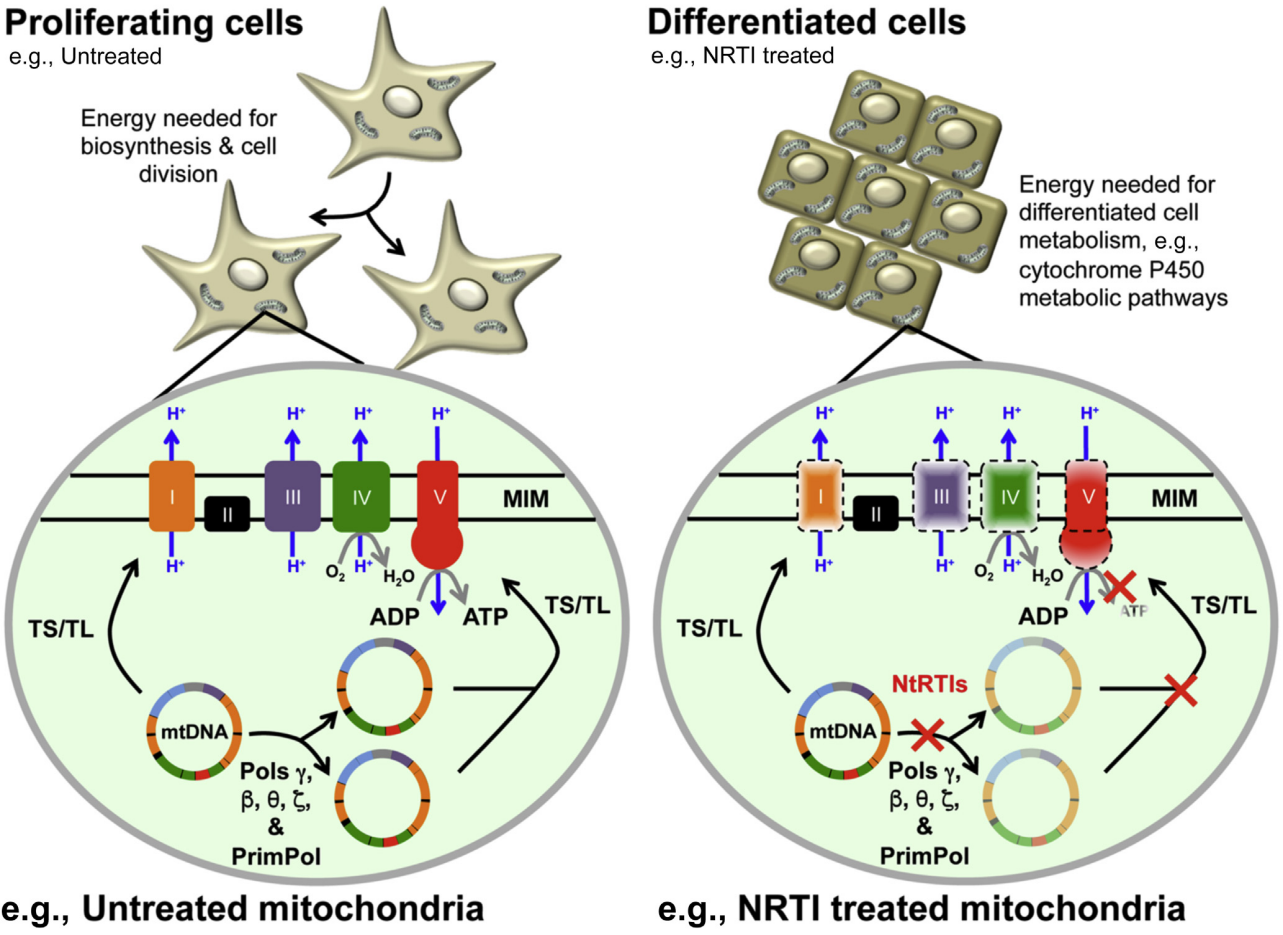
**Mitochondrial DNA (mtDNA) replication occurs in proliferating and differentiated cells.** Nucleoside reverse-transcriptase inhibitors (NRTIs) are metabolically activated by intracellular kinases to nucleotide reverse transcriptase inhibitors (NtRTIs). The insets highlight the mitochondrial matrix, the location of the DNA polymerases, and the mtDNA genome encoding 13 polypeptides of the oxidative phosphorylation (OXPHOS) machinery. The mtDNA genes are colored by OXPHOS complex, *orange*, *purple*, *green*, and *red*. *Orange*, OXPHOS complex I (NADH dehydrogenase); *black*, OXPHOS complex II (succinate dehydrogenase); *purple*, OXPHOS complex III (cytochrome bc1 complex); *green*, OXPHOS complex IV (cytochrome c oxidase); *red*, OXPHOS complex V (ATP synthase). The OXPHOS machinery is localized within the mitochondrial inner membrane (MIM). Within the mitochondrion, NtRTIs compete with native nucleotides at DNA polymerase active sites to inhibit mtDNA replication through chain termination and persistence in the mtDNA genome. H+, proton; O_2_, oxygen; H_2_O, water; ADP, adenosine diphosphate; ATP, adenosine triphosphate; TS, mitochondrial transcription; TL, mitochondrial translation; DNA polymerases gamma (Polγ), beta (Polβ), theta (Polθ), zeta (Polζ), and PrimPol. See the text for details.

Human cell culture studies have shown that exposure to ddC causes depletion of mtDNA-encoded OXPHOS subunits necessary for mitochondrial functions. In a 5-day ddC exposure study using PC-3 cells treated with different concentrations of the drug, exposure to 1 μM ddC caused a decline in the levels of the mtDNA-encoded COX1 protein down to 20% of vehicle control–treated samples. In contrast, the levels of the nDNA-encoded succinate dehydrogenase remained high at ∼85% of control-treated samples (60). Furthermore, Le Guillou *et al.* (61) recently demonstrated that a 2-week treatment of differentiated HepaRG with 20 μM ddC caused decreases in the levels of mtDNA and mtDNA-encoded ND1 and COX2 polypeptides but not in the amount of the nDNA-encoded COX4 polypeptide. Guillou *et al.* used quantitative PCR to detect differentiated HepaRG mtDNA levels that were reduced to ∼10% of untreated control levels, which is in good agreement with our results following 13 days of 12 μM ddC treatment, Figure 3*C*. Therefore, as mammalian mtDNA replication (and degradation/turnover) is independent of the cell cycle, the resulting ddC-induced bioenergetic impairments that we observed in differentiated (and undifferentiated) HepaRG likely result from insufficient mtDNA-encoded OXPHOS components, Figure 6. Maintaining the function of terminally differentiated cells in organs and tissues of patients chronically administered NRTIs is of concern as nonproliferating differentiated HepaRG suffered increased mtDNA depletion with increased treatment times; compare differentiated HepaRG mtDNA content in Figure 3, *B*–*C*. Although 13-day ddC treatments did not result in statistically significant differences in differentiated HepaRG cellular viability, proton leak–linked respiration was decreased by day 14. On the other hand, when differentiated HepaRG cells were treated with 0.25 μM ddC (and up to 128 μM ddC) for 15 days, cellular viabilities significantly decreased compared with vehicle control–treated cells, Figure 2*C* (*e.g.*, 78% and 52% viability for 1 and 8 μM ddC–treated cells respectively, *p* < 0.0001 for both by one-way ANOVA). Taken together with the observations that *1*) exposure to 12 μM ddC for 13 days caused less damage to proliferating cells as compared with a 15-day 8 μM ddC exposure (39% *versus* 6% viable cells remaining, respectively, compare Figures 4 and 2*B*) and that *2*) proliferating cell ATP-linked, basal, and nonmitochondrial respiration parameters were further altered/decreased at day 14 of ddC exposure as compared with day 8 (Fig. 5), we conclude that longer exposures to a mtDNA toxicant exacerbate mitochondrial damage in both proliferating and differentiated cells. An increased ddC exposure time would likely cause further mtDNA depletion and deterioration of bioenergetic functions in differentiated cells. The negative effects of long-term ddC treatment on postmitotic cell mitochondrial functions are particularly concerning for neuron and glia cells as ddC has been demonstrated to penetrate the blood–brain barrier in rats (49). Our results also raise concern regarding the health and regeneration of liver tissue when exposed to mitotoxic agents as the liver regeneration process has been proposed to involve both proliferating liver stem/progenitor cells and differentiated hepatocytes (62).

We found extreme defects in proliferating HepaRG cell mtDNA maintenance and bioenergetics associated with ddC exposure. After 6 days of ddC treatment, proliferating HepaRG mtDNA levels quickly plummeted to ≤2.5% of vehicle control–treated levels. Based on our quantitative analysis of mtDNA molecules per proliferating cell (2600, Fig. 1) and assuming a steady-state rate of mtDNA synthesis and degradation in cycling cells, we estimated that a level of 2.5% would be equal to 65 mtDNAs per cell. Assuming cell growth is not inhibited by ddC, and based on the calculated doubling time of 44 h (Fig. 2), if mtDNA molecules were simply depleted during cell division, then the levels would be ∼6.25% of the starting levels after ∼1 week of growth in ddC or 163 mtDNAs. A decrease to 6.25% is 2.5- and ∼4-fold higher than what was observed following 6 days of treatment with 1 and 12 μM ddC, respectively, Figure 3. Furthermore, proliferating cell growth was inhibited by exposure to 1 and 12 μM ddC as viable cell counts remaining on the culture dishes on day 7 were 73.4% and 55.8% of the control-treated cells, respectively, Figure 4. These data suggest that, in proliferating cells exposed to ddC, simple mtDNA dilution by cell division is not the only mechanism contributing to the loss of mtDNA. In this scenario, we predict that the mitochondrial degradosome plays an active role in mtDNA degradation in proliferating cells as it is in differentiated HepaRG. Perhaps the expression of degradosome subunits is upregulated in undifferentiated proliferating HepaRG causing enhanced mtDNA depletion relative to differentiated cells. By day 8 of ddC treatment, proliferating HepaRG mitochondrial bioenergetic failure was evident. Following 13 days of ddC exposure, mtDNA levels were ≤0.9% and growth and viability were significantly reduced. We predict that by this point in time the intrinsic apoptotic pathway has been activated. By day 14 of ddC exposure proliferating HepaRG basal and ATP-linked respiration parameters crashed, indicating severe mitochondrial bioenergetic failure.

Another explanation for exposure to ddC causing enhanced mtDNA depletion in proliferating HepaRG is that proliferating cells may have decreased drug resistance relative to differentiated cells. Differentiated HepaRG was demonstrated to express multidrug resistance–associated proteins including P-glycoprotein (*ABCB1*), MRP2 (*ABCC2*), and MRP3 (*ABCC3*), (63). These ATP-binding cassette (ABC) proteins function as ATP-dependent efflux pumps, which export drugs from the cell. In comparison with a CEM human lymphoblastoid cell line expressing low levels of P-glycoprotein, a CEM cell line expressing high levels of P-glycoprotein was demonstrated to be less effective at inhibiting HIV replication in the presence of AZT (64). Also, CEM cells that are resistant to the acyclic nucleoside phosphonate analog 9-(2-phosphonyl-methoxyethyl)adenine (PMEA, adefovir) were demonstrated to overexpress MRP4 (*ABCC4*), and MRP4 overexpression impaired the antiviral efficacy of PMEA and AZT-MP and correlated with the ATP-dependent efflux of the analogs (65). In a follow-up study, the PMEA-resistant cell line was used to show that MRP4 can mediate active cellular efflux of tenofovir in comparison with its PMEA-sensitive parental CEM cell line (66). In addition to the increased expression of degradosome gene products or downregulation of a ddC efflux pump, other possible mechanisms for enhanced mtDNA degradation in proliferating cells could include increased mitophagy (autophagic degradation of damaged mitochondria) or increased degradosome activity. We predict that lower concentrations of mitochondrial dNTPs in proliferating cell mitochondria could signal a switch between the p140 catalytic subunit DNA polymerase and exonuclease activities as *in vitro* the proofreading exonuclease activity of p140 degrades linear DNA in the absence of dNTPs (67). Whatever the mechanism, it is clear that future work is needed to understand how the molecular machinery regulating mtDNA homeostasis in proliferating and differentiated cells influences cellular susceptibility to mtDNA toxicants such as ddC.

In summary, we have shown that human proliferating and differentiated HepaRG cells are excellent model systems to study the harmful effects of a mitochondrial genotoxic agent, the antiretroviral ddC. The results of this study demonstrate the importance of carefully considering the off-target effects of a drug on both proliferating and differentiated cell mitochondrial functions. Caution needs to be taken when attempting to study drugs that inhibit proliferating cancer cell mtDNA replication as we have shown that nonproliferating differentiated HepaRG mtDNA genomes and mitochondrial bioenergetics are sensitive to ddC exposure. It is important to note that the mtDNA depletion observed in this study occurred in just under 2 weeks but patients treated with NRTIs are chronically exposed to drugs. We expect that the long-term exposure to mtDNA toxicants will be harmful to both proliferating and nonproliferating cell mitochondria *in vivo*. Awareness about the toxic effect of drugs and pollutants on mitochondrial function is rapidly accelerating (19, 68, 69, 70). For example, hepatitis C virus ribonucleoside analogs designed to inhibit viral RNA synthesis have been demonstrated to have off-target effects on the human mitochondrial RNA polymerase, POLRMT (71). POLRMT is a dual-purpose enzyme required for priming mtDNA replication and for carrying out mitochondrial-specific transcription. Another example is the polycyclic aromatic hydrocarbon benzo(a)pyrene, which causes more damage to mtDNA than to nDNA (20). Further complicating mitochondrial toxicity, genetic mutations encoding variants of DNA polymerase subunits, which localize to the mitochondrion, could predispose patients to mitochondrial toxicity, *e.g.*, p140 (R964C, R953C, and E1143D/G) and PrimPol D114N (72, 73, 74, 75, 76, 77). Although we have characterized the differential effects of a single NRTI on proliferating and differentiated HepaRG in this work, other antivirals, drugs, or environmental mitochondrial toxicants should be investigated in the future. As both proliferating and differentiated HepaRG are affected by ddC treatment, this study emphasizes that different cell types need to be taken into account when determining the off-target effects of environmental toxicants or drugs on mitochondrial function.

## Experimental procedures

### HepaRG cell culture and differentiation

The proliferating HepaRG hepatoma-derived cell line was purchased from Biopredic International (Saint-Grégoire, France). Cells were cultured and differentiated according to Biopredic International standard operating procedure and as described (25, 28, 31). Briefly, proliferating HepaRG were grown in Working Growth Medium (WGM) consisting of William′s E Medium (Thermo Fisher Scientific), 2 mM GlutaMAX (Thermo Fisher Scientific) with the addition of HepaRG Growth Medium Supplement (Lonza). Cells were grown at 37 °C and 5% CO_2_ in a humidified incubator, and the medium was refreshed every 3 days. For the day-to-day examination of cell culture, a Leica DMi1 inverted microscope with 10× and 20× phase contrast objectives was used. Proliferating HepaRG cells were passaged between days 12 and 15 post-seeding by washing with prewarmed Dulbecco′s phosphate-buffered saline (DPBS) followed by gentle trypsinization and neutralization with prewarmed WGM. Tissue culture dishes were seeded with 2 × 10^4^ proliferating cells/cm^2^ and cells were not passaged more than 18 times, P18. The Working Differentiation Medium (WDM) consisted of William′s E Medium (Thermo Fisher Scientific), 2 mM GlutaMAX (Thermo Fisher Scientific), with the addition of HepaRG Differentiation Medium Supplement (Lonza). The differentiation process was started 2 weeks after passaging proliferating HepaRG cells. The WGM was replaced with Combination Medium (CM), consisting of a 1:1 mixture of WGM to WDM, and 3 days later the CM was replaced with WDM. The medium was renewed every 3 days for 2 weeks and after 2 weeks cells attained differentiated hepatocyte-like morphology (see Fig. 1*A* for a timeline of HepaRG differentiation). Following treatment with prewarmed trypsin and neutralization with prewarmed medium, HepaRG viable cell counts were determined utilizing the trypan blue exclusion method.

### Determining ddC IC_50_ values

The HepaRG cytotoxic effect of ddC was initially estimated *via* percent growth inhibition relative to untreated control cells. This effect is expressed as the half-maximal inhibitory concentration (IC_50_) or concentration of ddC that reduces the number of viable treated cells by 50% relative to untreated control cells. The day before drug exposure, proliferating HepaRG cells were seeded at 2 × 10^4^ cells/cm^2^ in Falcon 24-well flat-bottom tissue culture plate wells and grown at 37 °C, 5% CO_2_ in a humidified incubator. On the next day, cells in triplicate wells were exposed to WGM treatment media containing 128, 8, 4, 2, 1, 0.5, 0.25, or 0 μM ddC for 15 days, and the treatment media were refreshed every 3 days. On the day of counting, treatment media were removed and the viable cells remaining in triplicate wells were washed with DPBS, trypsinized, neutralized with their respective treatment medium, and then counted using trypan blue. Untreated cell counts (0 μM ddC) were set to 100% and the IC_50_ values were calculated in Prism 7 using nonlinear regression analysis, the least-squares fit of inhibitor concentration *versus* normalized response. For the differentiated HepaRG experiment, proliferating HepaRG cells were seeded at 2 x10^4^ cells/cm^2^ in Falcon 24-well flat-bottom tissue culture plate wells and subjected to the differentiation protocol as described above. Following the differentiation process, cells in triplicate wells were exposed to WDM treatment media containing 128, 8, 4, 2, 1, 0.5, 0.25, or 0 μM ddC for 15 days, and treatment media were refreshed every 3 days. Viable differentiated cells were counted and IC_50_ values were calculated as described above for proliferating HepaRG.

### ddC treatment of proliferating HepaRG for whole-cell DNA extraction

Proliferating HepaRG in WGM were seeded at 2 × 10^4^ cells/cm^2^ and allowed to adhere to the bottom of cell culture dishes overnight at 37 °C, 5% CO_2_ in a humidified incubator. Treatment media were prepared by adding ddC to a final concentration of 0 (vehicle treated), 1, or 12 μM in WGM. On the next day, WGM from the cell culture dishes was replaced with WGM + vehicle, WGM + 1 μM ddC, or WGM + 12 μM ddC. Treatment media were refreshed every 3 days. On days 6 and 13 post-treatment two of each of the 0, 1, and 12 μM ddC–treated cell culture dishes were separately harvested (Fig. 3*A*). The medium was removed from each tissue culture dish to be harvested, and cells were gently washed with prewarmed DPBS followed by trypsinization and neutralization with their respective treatment media. The proliferating cells were completely suspended in treatment media + trypsin by gently pipetting up and down until the mixture appeared homogenous. The cells were transferred to conical tubes and a small aliquot was removed for counting. The cells were centrifuged at 250*g* for 5 min, washed in DPBS (6 ml per 1 × 10^7^ cells), and then resuspended in 2 ml DPBS per 1 × 10^7^ cells. One milliliter of the resuspension was added to each of two 1.5-ml microcentrifuge tubes. The tubes were centrifuged at 250*g* for 5 min at 4 °C, the supernatant was aspirated, and the cell pellets were stored at −80 °C. The experiment was performed three times. Whole-cell DNA was extracted from cell pellets as described below under “Whole-cell DNA extraction from HepaRG cell pellets.”

### ddC treatment of differentiated HepaRG for whole-cell DNA extraction

Proliferating HepaRG cells were differentiated as described above under “HepaRG cell culture and differentiation.” The WDM treatment media were prepared by adding ddC to a final concentration of 0, 1, or 12 μM. Cell culture dishes containing differentiated HepaRG were used to separately treat cells with 0, 1, or 12 μM ddC by replacing the WDM with WDM + vehicle, WDM + 1 μM ddC, or WDM + 12 μM ddC, respectively. The treatment media were refreshed every 3 days. On days 6 and 13 post-treatment two of each of the 0, 1, and 12 μM ddC–treated cell culture dishes were harvested (Fig. 3*A*). Treatment media were removed from each dish, and cells were washed with prewarmed DPBS followed by trypsinization and neutralization with their respective treatment media. The differentiated cells were completely suspended in the treatment media plus trypsin by gently pipetting up and down until the mixture appeared homogenous. Cells were counted, washed with DPBS, and frozen at −80 °C as described above for proliferating HepaRG. Whole-cell DNA was extracted from cell pellets as described below.

### ddC treatment of proliferating HepaRG for seeding Seahorse XFp miniplates

Proliferating HepaRG were seeded into tissue culture dishes and treated with ddC as described above under “ddC treatment of proliferating HepaRG for whole-cell DNA extraction.” On days 7 and 13 post treatment, two of each of the 0, 1, and 12 μM ddC–treated cell culture dishes were separately harvested, Seahorse cell culture miniplate wells were seeded, and bioenergetics was evaluated the following day using the Seahorse XFp Extracellular Flux Analyzer (Fig. 3*A*). On days of harvesting the cells, the treatment media were removed; cells were gently washed with prewarmed DPBS, trypsinized, neutralized with treatment media, and counted as described above. The suspensions of ddC-treated proliferating HepaRG were diluted down in treatment media such that the Seahorse cell culture miniplate wells were seeded with 1 × 10^4^ cells per well (9.4 × 10^4^ cells/cm^2^) in the treatment media (WGM +0, 1, or 12 μM ddC). The miniplates were incubated overnight at 37 °C, 5% CO_2_ in a humidified incubator followed by Seahorse extracellular flux analysis. The leftover cells that were not used to seed Seahorse miniplate wells were washed with DPBS, pelleted, and stored at −80 °C.

### ddC treatment of differentiated HepaRG for seeding Seahorse XFp miniplates

Proliferating HepaRG cells were differentiated and treated with ddC as described above in “ddC treatment of differentiated HepaRG for whole-cell DNA extraction.” On days 7 and 13 post-treatment, two of each of the 0, 1, and 12 μM ddC–treated cell culture dishes were harvested, cells were counted and then seeded into Seahorse cell culture miniplates as described above for proliferating HepaRG except that the WDM treatment media were used in place of WGM treatment media (Fig. 3*A*). Miniplates were incubated overnight at 37 °C, 5% CO_2_ in a humidified incubator followed by Seahorse extracellular flux analysis. The leftover cells that were not used to seed Seahorse miniplate wells were washed with DPBS, pelleted, and stored at −80 °C.

### Whole-cell DNA extraction from HepaRG cell pellets

Whole-cell DNA extracts were prepared based on the protocol developed by Kolesar *et al*. (78). Briefly, HepaRG proliferating or differentiated cell pellets were thawed and digested overnight at 55 °C in 500 μl proteinase K digestion buffer (100 mM Tris-Cl pH 8, 5 mM EDTA, 0.2% SDS, 200 mM NaCl, 0.3 mg/ml proteinase K, 1.1 mM 2-mercaptoethanol). The next morning, 10 μl of proteinase K digestion buffer was added to each sample, gently mixed, and incubated for 1 h at 55 °C. Cellular protein was removed from the samples by adding NaCl to a final concentration of 1.25 M, pelleting at 15,000*g* for 15 min, and collecting the supernatant into a fresh microcentrifuge tube. The total nucleic acid was precipitated from the supernatant *via* ethanol precipitation. The pellets were dried away from light for ∼1 h then resuspended in 1× TE buffer (10 mM Tris-Cl, pH 8.0, 1 mM EDTA) plus 1 mM dimethyl urea. Samples were stored in the absence of light at room temperature overnight then frozen at −20 °C. The whole-cell DNA concentrations were measured in ≥triplicate using a Qubit fluorometer (Thermo Fisher Scientific) according to the manufacturer’s specifications.

### Synthesis of digoxigenin-labeled probes, Southern blotting, and immunodetection of target DNA fragments

Nuclear DNA (nDNA)- and mitochondrial DNA (mtDNA)-specific probes used for Southern blotting and immunodetection of target DNA fragments were synthesized *via* PCR using the PCR DIG Probe Synthesis Kit (Roche) as described (79). Briefly, each probe was synthesized separately from their respective plasmid templates. The pCR2.1-TOPO-18S plasmid harbors a cloned fragment of the human nuclear 18S ribosomal pseudogene 4 (RNA18SP4), and the pCR2.1-TOPO-mtDNA plasmid harbors a cloned fragment of the human mtDNA sequence from nucleotide positions 168 to 604. Twenty-five-microliter PCR reactions for each probe contained 50 pg plasmid, 1× PCR buffer with 1.5 mM MgCl_2,_ 0.5 μM of each respective primer, 1× PCR DIG Probe Synthesis mix, and 52.5 mU/μl of Expand High Fidelity Enzyme. 18S nDNA probe reactions contained 200 μM dATP, dCTP, and dGTP, as well as 130 μM dTTP and 70 μM DIG-11-dUTP. When synthesizing the mtDNA probe it is necessary to use a final DIG-11-dUTP concentration of 35 μM owing to the high GC content in pCR2.1-TOPO-mtDNA. In addition, the mtDNA probe reactions contained 200 μM dATP, dCTP, and dGTP, as well as 165 μM dTTP. PCR Thermocycling conditions were: initial denaturation for 2 min at 95 °C, 32 cycles of denaturing for 30 s at 95 °C, annealing for 30 s at 60 °C, and extension for 40 s at 72 °C. A final extension for 4 min at 72 °C was carried out. DIG-labeled probes were heated by boiling in a water bath for 5 min followed by rapid cooling on ice. Two microliters of heat-denatured mtDNA probe and 4 μl of 18S nDNA were added per milliliter of prehybridization solution (Roche) prewarmed to 50 °C for simultaneous detection of both probed sequences in BamHI-digested whole-cell DNA extracts. The probe mixture was used immediately or stored at −20 °C until needed.

One microgram of whole-cell extracted DNA was digested with 5 units of BamHI (Thermo Scientific) in a 1× digestion buffer for 3 h at 37 °C. The digested samples were loaded on to 1.0% agarose gels in 1× TAE buffer (40 mM Tris, 20 mM acetic acid, 1 mM EDTA) without ethidium bromide and were electrophoresed at 20 V for 16 h. Gels were soaked in 0.25 N HCl for 5 min to partially depurinate the DNA. Next, gels were rinsed in sterile Milli-Q water, soaked twice in denaturing solution (0.5 N NaOH, 1.5 M NaCl) for 15 min, rinsed in autoclaved Milli-Q water, and then soaked twice in neutralizing solution (0.5 M Tris-HCl, pH 7.5, 3 M NaCl) for 15 min. These steps allow for in-gel DNA denaturation and fragmentation of high-molecular-weight DNA molecules (*e.g.*, the 16.6-kb mtDNA) into smaller pieces to allow efficient transfer to the membrane during Southern blotting. An electrophoresis gel tray was placed upside down inside a container filled halfway up the gel tray with 20× SSC buffer (3 M NaCl, 0.3 M sodium citrate, pH 7.0). A long piece of 20× SSC-soaked Whatman paper was placed on top of the gel tray with ends angled down into the 20× SSC buffer to act as a wick. The gel was rinsed once more in water then placed on top of the wick. A piece of Amersham Hybond – N^+^ positively charged membrane (GE Healthcare Life Sciences) cut slightly larger than the gel was soaked in 2× SSC buffer for 1 min then placed on top of the gel. Two pieces of Whatman paper cut the same size as the membrane were soaked in 2× SSC then placed on top of the membrane and a test tube was used to roll out any bubbles. An ∼5-cm stack of paper towels cut slightly smaller than membrane was placed on top of Whatman papers and a 200-g weight was used to allow the capillary transfer of DNA to the membrane overnight. The Southern blot was disassembled and all paper towels were discarded. The membrane was placed onto a Fotodyne transilluminator and exposed to UV light for 2 min to cross-link DNA onto it. The membrane was rinsed in water and allowed to air dry.

Southern blot membranes were placed into 35 × 150-mm hybridization bottles with 6 ml of prehybridization solution (Roche) and incubated in a hybridization oven for 2 to 3 h at 50 °C. The prehybridization solution was discarded and replaced with the dual probe solution then incubated at 50 °C overnight. On the next day, probe solutions were removed and membranes were washed twice in 2× SSC + 0.1% SDS for 5 min at room temperature in the hybridization oven. Next, the blots were washed twice with prewarmed 0.5× SSC + 0.1% SDS at 65 °C for 15 min. The membranes were removed from hybridization bottles and equilibrated in a maleic acid buffer (0.1 M maleic acid, 0.15 M NaCl, pH 7.5) for 2 min by mixing on an orbital shaker at 50 rpm then placed in 40 ml 1× blocking buffer (Roche) + 0.015% SDS and mixed for 30 min. The blocking buffer was made by adding 100 ml of 10× blocking buffer (Roche) to 900 ml of maleic acid buffer. The anti-DIG alkaline phosphatase conjugate (Roche) was added to the blocking solution + 0.015% SDS at 75 mU/ml and membranes were incubated for ∼1 h with mixing. The blocking solution containing the antibody was discarded, the membrane was rinsed three times with Milli-Q water, then the membrane was washed twice with wash buffer (maleic acid buffer, 0.3% Tween20, 0.015% SDS) by mixing for 15 min. The wash buffer was discarded and replaced with the detection buffer (0.1 M Tris-HCl, 0.1 M NaCl, pH 9.5) for 3 min. The CDP-star solution was made by mixing 10 μl of 25 mM CDP-Star (Roche) with 0.99 ml of detection buffer in a microcentrifuge tube. The membrane was removed from the detection buffer and placed on clear plastic wrap. The CDP-star substrate was evenly distributed onto the membrane and incubated for 1 min at room temperature. The membrane was then placed on another piece of plastic wrap, and the plastic wrap was folded over the membrane to keep it wet. The membrane was incubated at 37 °C for 5 min then imaged using Syngene G:Box.

### Estimation of mtDNA copy number utilizing a pCR2.1-TOPO-mtDNA standard curve

Cell counts, DNA concentrations, and Southern blot data were utilized to estimate the mtDNA copy number. Standard curves were generated using a series of dilutions of the pCR2.1-TOPO-mtDNA plasmid, the same plasmid that was used to synthesize the DIG-labeled mtDNA probe. The pCR2.1-TOPO-mtDNA plasmid harbors a cloned fragment of the human HEK-293 (ATCC CRL-1573) mtDNA sequence from nucleotide positions 168 to 604, and this sequence differs from the NC_012920.1 reference sequence at three positions: 263 A>G, 309_310insC, and 315_316insC. Digestion with HindIII that cuts the plasmid once upstream of the mtDNA sequence in the polylinker linearized pCR2.1-TOPO-mtDNA. The plasmid was isolated using the Omega Bio-Tek E.Z.N.A. Plasmid DNA Mini Kit II. The plasmid DNA was digested with 5 U/μg of HindIII for 3 h at 37 °C. Next, the linearized plasmid was run on an agarose gel for 1 h at 100 V, the plasmid bands were cut out of the gel, and the gel was extracted using the Omega Bio-Tek E.Z.N.A. Gel Extraction Kit. The gel extracted plasmid DNA concentration was measured in triplicate using a Qubit fluorometer. The plasmid was diluted down to 0.696 ng/μl, then the plasmid concentration was measured again in triplicate. Ten microliters of 0.696 ng/μl plasmid was loaded onto gels along with 10 μl of two 2-fold serially diluted samples, 0.348 and 0.174 ng/μl. We assume that the probe will hybridize to the plasmid and the mtDNA sequences identically. One microgram of WCE DNA samples was loaded along with the plasmid samples, and linear regression analysis was utilized to estimate the copies of differentiated HepaRG mtDNA in each 1 μg WCE DNA sample. As the 4.4-kb plasmid and the 16.6-kb mtDNA differ in size by 12.2-kb, 1 ng of plasmid contains the same number of molecules as 3.8 ng of mtDNA.

### Seahorse XFp bioenergetic analysis

The Seahorse XFp extracellular flux analyzer was used to measure bioenergetics as previously described (31). Briefly, 1 × 10^4^ proliferating or differentiated HepaRG cells were treated sequentially with 2 μM oligomycin, 1 μM carbonyl cyanide p-trifluoromethoxy-phenylhydrazone (FCCP), and 0.5 μM antimycin A + 0.5 μM rotenone (all purchased from Fisher Scientific). After the experiments, cells were gently washed with 200 μl prewarmed DPBS, incubated overnight at −80 °C, then lysed in ice-cold RIPA lysis buffer (50 mM Tris-Cl pH 8.0, 150 mM NaCl, 1% Igepal CA-630, 0.5% deoxycholate, 0.1% sodium dodecyl sulfate) supplemented with a 1 in 101 dilution of HALT protease inhibitor cocktail (Thermo Fisher Scientific). The total cellular protein content in each miniplate well was measured using the Pierce bicinchoninic acid protein assay kit (Thermo Scientific), and OCR and ECAR values were normalized to total cellular protein. Data were obtained from three independent experiments performed on different days and are presented as mean ± standard deviation (SD).

### Equations used to calculate mitochondrial respiration parameters

Mitochondrial respiration parameters were calculated as follows: *I. Basal respiration* = (Last rate before the first injection) − (Nonmitochondrial respiration rate), *II. Proton leak* = (Minimum rate after oligomycin injection) − (Nonmitochondrial respiration rate), *III. Maximal respiratory capacity* = (Maximum rate after FCCP injection) − (Nonmitochondrial respiration rate), *IV. Spare respiratory capacity or reserve respiratory capacity* = (Maximal respiratory capacity) − (Basal respiration), *V. Nonmitochondrial respiration* = Minimum rate after rotenone + antimycin A injection, *VI. ATP-linked respiration* = (Last rate before oligomycin injection) − (Minimum rate after oligomycin injection) (53).

### Statistical analyses used

For the analysis of bioenergetics data, statistical analysis was run using a three-way ANOVA (*i.e.*, 2 × 2 × 3) with follow-up post hoc tests to determine significance. We examined the cell type (proliferating or differentiated), day (8 or 14), and concentration (0, 1, and 12 μM ddC) for each variable of interest. SAS software version 9.4 was used for data analysis. For analyses of all other data, statistical significance between two parametric groups with unequal variance was determined using a Welch’s *t* test. Comparisons of more than two groups of parametric data were assessed by one-way analysis of variance (ANOVA) followed by Tukey’s test or Welch’s ANOVA with Games–Howell post hoc. Comparisons of greater than two groups of nonparametric data were assessed by Kruskal–Wallis tests followed by Dunn’s post hoc test. *p*-values less than 0.05 were considered significant.

## Data availability

All of the data described herein is within this article. Reagents, plasmids, etc., are available upon request by contacting the corresponding author M. J. Y.

## Conflict of interest

The authors declare that they have no conflicts of interest with the contents of this article.
