# Supplementary material for: The antiretroviral 2′,3′-dideoxycytidine causes mitochondrial dysfunction in proliferating and differentiated HepaRG human cell cultures
Source: J Biol Chem. 2020 Dec 31;296:100206. doi: 10.1074/jbc.RA120.014885 (PMC7948951; doi:10.1074/jbc.RA120.014885)
Supplement: Figure S1 [file mmc1.pdf]

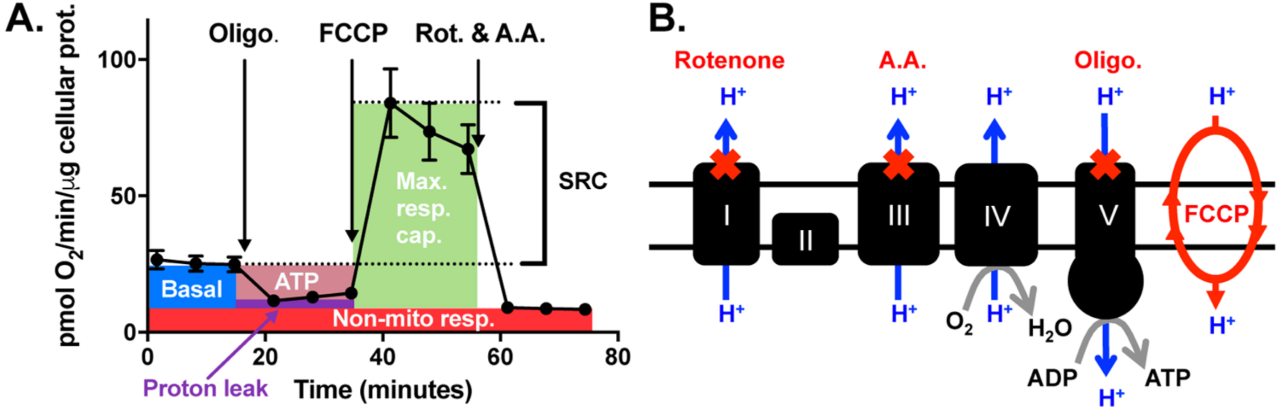

Supplementary Figure 1. Mito Stress test bioenergetic parameters. **A.** Mitochondrial bioenergetic parameters: basal respiration (Basal), ATP-linked respiration (ATP), maximal respiratory capacity (Max. resp. cap.), spare respiratory capacity (SRC), proton leak, and non-mitochondrial respiration (Non-mito resp.). Metabolic stressors are injected sequentially from Ports a (2 μM oligomycin final well concentration), b (1 μM FCCP final well concentration), and c (0.5 μM antimycin A + 0.5 μM rotenone, final well concentrations). Olig., oligomycin; Rot., rotenone; A.A., antimycin A. **B.** Cartoon of the mitochondrial inner membrane OXPHOS machinery targeted by key stressors used in the Mito Stress test.
